# The change of definitions in a multidisciplinary landscape: the case of human embryo and pre-embryo identification

**DOI:** 10.3325/cmj.2016.57.510

**Published:** 2016-10

**Authors:** Cinzia Piciocchi, Lucia Martinelli

**Affiliations:** 1 Faculty of Law, University of Trento, Trento, Italy; 2 MUSE – Science Museum, Trento, Italy

*“A word is dead When it is said, Some say. I say it just begins to live that day”* Emily Dickinson

In contemporary knowledge landscapes, interdisciplinary communication is crucial. In many cases, the interaction among experts of different fields is challenging, especially with regard to controversial definitions at the intersection of law, science, and bioethics. We analyze the legal definitions of the term “embryo” as the result of legal, ethical, as well as scientific implications, at the intertwining of social changes and scientific advancements. We show how embryo is a complex word, which may be interpreted in different ways. Taking into account the latest research advances on early stage embryo development, we focus on the delicate roles of law in balancing differing claims of scientific investigation and bio-ethic concerns. We conclude that sensitive ethical definitions are the synthesis of often competing interests among different disciplines. Moreover, human feelings toward controversial biology innovations should be taken into account.

## Accurate definitions for attentive identifications

Definitions are challenging. According to Oxford Dictionaries, the term “definition” is intended as “a statement of the exact meaning of a word” whose function is to provide “an exact statement or description of the nature, scope, or meaning of something” (*http://www.oxforddictionaries.com/definition/english/definition*). Definitions, however, are based on words, and words are also tricky issues since, as poetically pointed out by Emily Dickinson (1): the life of words is a mysterious matter. Their sounds, spelling, and meaning may change radically over time and sometimes it is necessary to look back at their roots, in search of a “true” sense. In some cases, they come up to define newness and start to live right before our very eyes. Even during their existence, words assume different meanings, according to various contexts. Hence, the same word might have different meanings for different actors of a society.

For lawyers, definitions are crucial. Law deals with human behaviors, subsuming facts and human conducts under pertinent norms. In criminal law, for example, the same behavior is defined as murder, or manslaughter, or no crime at all depending on the circumstances like intention or self-defense, competency, etc. In tort law, liability depends on the existence of a damage, but it occurs if the behavior is classified as negligence and there is a causal relation between conduct and harm. Definitions are also important when law deals with science. The legal definition of death, for example, changed over time from cardio-pulmonary to brain death criteria. Nothing changed in the dying process, but shifting definition was needed to allow organ transplants (2).

In science, definitions are expected to express contemporary knowledge in the most specific way, so that, as already stated “by reading the definition only, it should ideally not be possible to refer to any other entity than the definiendum” (3). Despite this assumption, within the scientific community, efforts to formulate unequivocal definitions are challenging and often controversial. This is the case, for instance, for the term “life,” for which many definitions have been attempted in various disciplines, but consensus is still lacking (4). Moreover, even within the same area of research, when common techniques are employed in different fields of specializations, reciprocal use of terminology is often misused. Misleading and inconsistent use of key words and lack of unified vocabulary have been pointed out in scientific literature (5). There is a clear need to adopt a more consistent application of terminology, to prevent confusion and to improve communication among scientists, as it has been pointed out, for instance, by the Tissue Culture Association (6) and in the field of immunology and cancer biology (7).

In the case of biology innovations, some of them referred to as bio-objects (8), definitions are problematic, being the result of the interplay between natural sciences and humanities, such as life sciences, ethics, and law. It should not be taken for granted that the same term would have the same meaning for a scientist, a bioethicist, and a judge. Properly defining various bio-objects would be beneficial, and it is yet challenging since their identity may even mutate in different contexts (9). Thus, in this interdisciplinary framework, communication is particularly challenging, and analyzing words and definitions becomes a remarkable and interesting topic as well as a must in the current multidisciplinary knowledge landscape. In the terminology adopted for describing transgenics, for instance, a conscious use of vocabulary may communicate technical information or deliver subjective evaluation. Thus, while the more technical expression “gene transfer” would describe the process of integrating genes from a genome to another, the commonly used (also in scientific literature) “genetic transformation” would emphasize the concept of “changing nature,” which – worth stressing – has been proved to be a quite distasteful feeling for citizens-consumers (10). Moreover, a wise adoption of terms could have impact on regulation. A paradigmatic case is the hot debate, which has been recently involving scientific community, policy makers, and environmentalists, on the proper denominations of “cis-genic” plants, being these plants the result of modifications involving (a) natural gene(s) belonging to conventional breeder’s gene pool (11). The adoption of a more precise definition is aimed at identifying such plants as different from the “trans-genics,” which contain foreign genes instead, and at introducing for them an alternative European regulatory regime (or even de-regulate them) than the one already applied for the “trans-genic” ones (11).

In medicine, Assisted Reproduction Technologies (ARTs), shifting the attention from a medical procedure to social relations, offer interesting cases for analyzing the science discourse and its implication in regulation of science innovations (12). The human embryo definition, as product of ARTs, is an interesting example of this challenging debate. This same bio-object is variously defined by different national laws, and even different definitions are sometimes adopted within the same law, with regard to its stage of development or to its end use. As focused below, a human embryo, which from the scientific point of view is identified according to objective features, may assume different identities according to its fate, ie, whether it is to be implanted to develop a human being, or to be used in research, or to be stored in bio-banks, to be exchanged, or to be the left-over of a laboratory.

The various possible definitions of the concept of “embryo” and the complexity of adopting suitable terminology as an outcome of the development of modern biotechnology have been previously analyzed (13). Taking in consideration the recent scientific advancements in human embryology, growing *in vitro* embryos for longer than ever before, up to 12-13 days (14,15), we considered the legal definition of human embryo as the result of a controversial dialogue among science, bio-ethics, and social issues. To this aim we analyzed why a meticulous definition of specific steps of embryo development was important in the decision-making debate on the use of human embryo for research purposes.

## Adapting distinct terms to adopt a suitable regulation

During the 1970s, when the Constitutional Courts of various countries, especially in the US and in Europe, had to face embryo’s legal status with regard to abortion legalization, embryo was obviously intended as the encounter of ovum and sperm occurring in the body of a woman. The focus, in those cases, was the balancing of embryo’s and mother’s conflicting rights. In the leading case on abortion *Roe v. Wade*, for example, the US Supreme court provided a historical perspective on “the question in terms of the point at which the embryo or fetus became ‘formed’ or recognizably human”, by focusing on the question of “when a ‘person’ came into being” (see *Roe v. Wade*, 410 US 113, 1973), ie, a question which biology is not deputed to solve. The further capability to generate *in vitro* embryos started a process of bio-objectification well analyzed by Beriain (13), giving rise to a whole series of questions concerning “embryo” definition when its existence starts outside a female body as well as boundaries of its use. Following the first baby conceived with *in vitro* fertilization in 1978, in the UK, the Warnock committee (as named after its chairwoman, Baroness Mary Warnock) was established to inquire into the ethical, scientific, and legal implications of ARTs.

For the purpose to consider multiplicity of views and social rather than individual issues, the works of the committee may be seen as an effort to care for the impact of a biology innovation on society and to apply medical innovation with a responsible approach. Of the whole debate, the possibility to use human embryos in research arose as one of the most sensitive issues. Thus, the committee, even never using the term “pre-embryo,” in its final report “Inquiry into Human Fertilization and Embryology” released in July 1984, laid the foundations of the notion of timing for indicating the stages of development, which had to be considered with regard to the regulation of *in vitro* embryos for research purposes (*http://www.hfea.gov.uk/docs/Warnock_Report_of_the_Committee_of_Inquiry_into_Human_Fertilisation_and_Embryology_1984.pdf*). Accordingly, “the meeting of egg and sperm at fertilization” was taken as a starting point and “the ensuing six weeks immediately following fertilization” were regarded as embryonic stage. Thus, the report, by fixing a start and by delimiting a time period, made clear that although no biological reasons claimed to set a limit to the development of the *in vitro* embryo, decisions were to be taken “in order to allay public anxiety” by ensuring that “embryos should not be kept alive for an undefined period.” The committee reported various proposals, determining at 17 days the deadline of *in vitro* embryo development, when “neural development begins,” or “at the end of implantation stage.” It finally took as reference point the formation of the primitive streak, ie, a well characterized histological structure formed at one end of the embryonic disc on the fourteenth or fifteenth day after fertilization, which marks the beginning of embryo’s head-to-tail axis (16). Appearance of the primitive streak indicates the setting of the embryo body plan, which was not yet present in the previous developmental stages. Pointing out that “this structure marks the beginning of individual development of the embryo,” the committee recommended that embryo research should be carried up to 14 days after fertilization.

Such same limit has been also indicated by the American Ethics Advisory Board (EAB) in 1979 (*https://repository.library.georgetown.edu/bitstream/handle/10822/559350/HEW_IVF_report.pdf?sequence=1*)*,* which used for this stage of embryo development various definitions like “early human embryo” or “preimplantation embryo” with reference to “the completion of implantation” of embryos into the uterus. The EAB concluded that: “… no embryos will be sustained *in vitro* beyond the stage normally associated with the completion of implantation (14 days after fertilization)”. The 14-day limit has been subsequently adopted in the reports of other ethical boards, in various countries, such as Australia and Canada (17).

The attempt to properly identify this early stage of embryo development resulted in the rise of the term “pre-embryo,” which was introduced by the mammalian developmental biologist, also involved in ethical issues, Clifford Grobstein in the paper “External Human Fertilization,” published in *Scientific American* in 1979 (18,19). The scientific threshold evocated to support the 14-day development was the twinning potentiality up to this timing. Moreover, Grobstein’s use of the synonym “pre-human” and his referring to the moral status of the pre-embryo alike that one of cells and tissues may be interpreted as an attempt to support a moral matter with biology. Since its first adoption, this term has been extensively debated and variously – often critically – regarded as “a new metabiological concept” (17), a “humpty dumpty word” (as during the UK parliamentary debate regarding the unborn children protection of 1986 (*http://hansard.millbanksystems.com/commons/1986/oct/21/unborn-children-protection-no-2-bill*), a “rethorical device” (20) or a “reductivist definition” (21).

The need to use in bio-law a terminology to suitably define this stage of embryo development is documented by the various locutions used to define pre-embryo. An interesting example is the legal definition in the Spanish law regulating ARTs, adopted in 2006, whose article 1 stated that a preembryo (“*preembrión”*) has to be the intended *in vitro* embryo derived from the group of cells resulting from the progressive division of the oocyte until 14 days after fertilization. Again, the law established a 14-day limit for research on embryos (art. 15 of the Law n.14/2006, about Assisted Reproductive Techniques) but, as well pointed out by scholars, in this phrasing, pre-embryo was defined first of all as an “embryo,” which is further characterized by its stage of development (22). In 1999, the Constitutional Court of Spain (decision n. 116/1999) used the term “*preembrión”* – besides other expressions like the fruit of conception (“*fruto de la concepción*”) – and stated that *in vitro* pre-embryos did not enjoy the same constitutional protection as the ones already transferred *in utero*. Other laws do not use the term pre-embryo, but clearly refer to it, as in the case of the Greek regulation statute (law n. 3089/ 2002 Medically Assisted Human Reproduction, *http://policy.mofcom.gov.cn/GlobalLaw/*), which, regulating the use of surplus “cryopreserved reproductive material” and of “non cryopreserved fertilized ova,” points out that they are to be destroyed after the completion of 14-day postfertilization.

Despite the above discussed attempt to apply proper definitions, in other laws, even in those regulating embryo research up to 14 days, no other terms but “embryo” are used. Worth stressing, this also occurs in laws prohibiting this research, such as in the Italian law, which uses the term “*embrione*” *tout court* (law n. 40/2004 on ART) and in the Swiss law (LRCS, 810.31, of 19 December 2003, The Federal Act on Research Involving Embryonic Stem Cells), which regulates the permission to undertake embryo stem cell research and adopts the term “*embryo*” in the German version and the corresponding terms “*embryon*” and “*embrione*” in the French and Italian versions respectively. This latter law, on the basis of biology and of applications, also details what has to be considered as an embryo, a spare embryo, embryo stem cells, and also a parthenote.

Finally, in this analysis of terminology, it must be taken into account that the term “embryo” as defined by laws regulating ARTs might be intended differently as compared with the “embryo” as considered by Supreme Courts dealing with abortion, or with the embryo as defined by the European Court of Justice (ECJ), to the extent of patenting rights. Indeed, EU law excludes from patentability the use of “human embryos for industrial or commercial purposes.” Worth stressing, the interpretation of what should be intended as human embryo has been focused on a specific feature, such as the inherent potentiality to become a human being, rather than on the final product of its manipulation, ie, the embryo. In a first decision, in fact, (*Oliver Brüstle v Greenpeace* e.V., C-34/10, 18 October 2011), the Court included in the definition of embryo various results of egg manipulation, such as “any human ovum after fertilisation, any non-fertilised human ovum into which the cell nucleus from a mature human cell has been transplanted, and any non-fertilised human ovum whose division and further development have been stimulated by parthenogenesis” (*Oliver Brüstle v Greenpeace*, 2011, *http://curia.europa.eu/juris/document/document.jsf?text=&docid=115334&pageIndex=0&doclang=EN&mode=lst&dir=&occ=first&part=1&cid=59273*). In 2014, the Court of Justice gave a partially different definition, ruling that: “(…) an unfertilised human ovum whose division and further development have been stimulated by parthenogenesis does not constitute a ‘human embryo’ (…) if, in the light of current scientific knowledge, it does not, in itself, have the inherent capacity of developing into a human being (…)”. (International Stem Cell Corporation v Comptroller General of Patents*,* 2014, *http://eur-lex.europa.eu/legal-content/EN/TXT/?uri=CELEX%3A62013CJ0364*). The ECJ took as a starting point respectively the definition of the “concepts of ‘human embryo’ and ‘use for industrial or commercial purposes’” (*Brüstle*) and the “concepts of ‘human embryo’ and ‘organism capable of commencing the process of development of a human being’” (*International Stem Cell Corporation*). These two decisions are both relevant in the debate concerning the intellectual property field and once more they suggest, in embryology application, the recurrent need to establish the start of “being human.”

It must be remarked that on May 2016, the International Society for Stem Cell Research (ISSCR) published its revised and updated guidelines for stem cell research and clinical translation, adopting the 14-day limit to define prohibited research activities on human embryos (*http://www.isscr.org/home/publications/2016-guidelines*). This prohibition was based on 3 arguments, considering that these experiments “lack a compelling scientific rationale, raise substantial ethical concerns, and/or are illegal in many jurisdictions.” The same month (May 2016), on the other hand, *Nature* (14) and *Nature Cell Biology* (15) reported that scientists had grown in vitro intact embryos for longer than ever before: up to 12-13 days, stopping the study in accordance with the 14-day limit of law and international guidelines. Shedding light on mysterious key events of early human morphogenesis, including structural features, gene expression, and species-specificity of early developmental events, this achievement is proposed as an important enhancement of human embryology, which also opens new perspectives on medical aspects of pregnancy, ARTs, and use of animal models in human research. Accordingly, a debate about the possibility to extend the 14-day limit is likely to be expected (23,24). These challenging results show once more the often conflicting needs of scientific investigation and bio-ethic concerns and the delicate role of law in balancing their differing claims.

Finally, it should also be remembered that words are used not only to describe facts, but also to communicate perceptions. The definition of embryos to the extent of research regulation is a complicated issue, as previously well analyzed (25). Law needs biology to understand reality, but feelings toward morally charged entities – like both natural and ART embryos are – are quite relevant in the process of defining embryo legal status. The variety of definitions surrounding cell development after fertilization adopted by national laws does not describe different bio-objects but rather shows different views. For this reason those definitions are the result of different elements, such as biology evidence, ethical considerations, legal issues and, worth stressing, empathy, ie, a key component defined, in 2002, as emotional involvement (“*investissement émotionnel*”) by the Belgian Advisory Committee on Bioethics (*http://www.health.belgium.be/en/belgian-advisory-committee-bioethics*). Accordingly, besides biology, human feelings intermingle into legal definitions with a dynamic and controversial process continuously negotiated between scientific evidence and human emotions and values.

## Conclusions

In science, new terms have been conceived following discoveries or to define novel products of innovations. Moreover, as well shown in ARTs, definitions seem to be fluid matters being subjected to continuous re-interpretation when facing demands of lay-society, needs of moral decision-making, and in different contexts. In law, the attempt to clearly identify bio-objects and develop proper regulations results in a variety of descriptions and interpretations according to socio-cultural landscapes and their evolutions/involutions. In the case of embryo, for instance, if its development is continuous from the biological perspective, law requires fixing specific features, which would fit regulation needs rather than strictly represent scientific facts. This is the case of legal interpretation of the term “pre-embryo,” for which the 14-day development period seems to have a strong symbolic and moral value. At the same time, however, such limit might result in an adaptable parameter since legal definitions are subjected to changes in the dialogue with science and ethics.

Only through the understanding of this complexity, a truly interdisciplinary approach is possible. In this demanding exercise, different experts are claimed to cooperatively interchange their specific skills to build up a knowledge landscape where different visions blend to regulate social and personal needs, opportunities, and limits.
